# A Randomized Placebo-Controlled Study of a Transcranial Photobiomodulation Helmet in Parkinson’s Disease: Post-Hoc Analysis of Motor Outcomes

**DOI:** 10.3390/jcm12082846

**Published:** 2023-04-13

**Authors:** Claire McGee, Ann Liebert, Brian Bicknell, Vincent Pang, Vivian Isaac, Craig S. McLachlan, Hosen Kiat, Geoffrey Herkes

**Affiliations:** 1 Faculty of Health Sciences, Torrens University Australia, Sydney, NSW 2000, Australia; claire.mcgee@torrens.edu.au; 2 School of Medical Sciences, University of Sydney, Camperdown, NSW 2050, Australia; ann.liebert@outlook.com; 3 Department of Research and Governance, Sydney Adventist Hospital, Wahroonga, NSW 2076, Australia; 4 NICM Health Research Institute, University of Western Sydney, Westmead, NSW 2145, Australia; brian.bicknell@pbmconsults.com (B.B.); vincent.pang87@gmail.com (V.P.); hosen.kiat@chi.org.au (H.K.); 5 School of Allied Health, Exercise & Sports Sciences, Faculty of Science & Health, Charles Sturt University, Albury Campus, Albury, NSW 2640, Australia; anisaac@csu.edu.au; 6 Centre for Healthy Futures, Torrens University Australia, Sydney, NSW 2000, Australia; craig.mclachlan@torrens.edu.au; 7 Faculty of Medicine, Human and Health Sciences, Macquarie University, Sydney, NSW 2109, Australia; 8 College of Health and Medicine, Australian National University, Canberra, ACT 2601, Australia; 9 Cardiac Health Institute, Sydney, NSW 2000, Australia; 10 Department of Neurology, Sydney Adventist Hospital, Wahroonga, NSW 2076, Australia

**Keywords:** photobiomodulation, transcranial, Parkinson’s disease, mobility

## Abstract

Emerging evidence is increasingly supporting the use of transcranial photobiomodulation (tPBM) to improve symptoms of neurodegenerative diseases, including Parkinson’s disease (PD). The objective of this study was to analyse the safety and efficacy of tPBM for PD motor symptoms. The study was a triple blind, randomized placebo-controlled trial with 40 idiopathic PD patients receiving either active tPBM (635 nm plus 810 nm LEDs) or sham tPBM for 24 min per day (56.88J), six days per week, for 12 weeks. The primary outcome measures were treatment safety and a 37-item MDS-UPDRS-III (motor domain) assessed at baseline and 12 weeks. Individual MDS-UPDRS-III items were clustered into sub-score domains (facial, upper-limb, lower-limb, gait, and tremor). The treatment produced no safety concerns or adverse events, apart from occasional temporary and minor dizziness. There was no significant difference in total MDS-UPDRS-III scores between groups, presumably due to the placebo effect. Additional analyses demonstrated that facial and lower-limb sub-scores significantly improved with active treatment, while gait and lower-limb sub-scores significantly improved with sham treatment. Approximately 70% of participants responded to active treatment (≥5 decrease in MDS-UPDRS-III score) and improved in all sub-scores, while sham responders improved in lower-limb sub-scores only. tPBM appears to be a safe treatment and improved several PD motor symptoms in patients that responded to treatment. tPBM is proving to be increasingly attractive as a possible non-pharmaceutical adjunct therapy.

## 1. Introduction

Parkinson’s disease (PD) is the second most common neurodegenerative disorder with a complex pathogenesis that results in a heterogenous mix of motor and non-motor manifestations [1]. Due to the increasing prevalence of PD in the past 20 years and its progressive nature, the effects of PD on disability-adjusted life years is significant and expected to increase [2,3,4,5].

There are a wide variety of treatment approaches that minimize symptoms and improve quality of life. For example, dopamine supplementation can improve both quality of life and motor symptoms [6], and deep brain stimulation offers significant improvements in motor symptoms [7]. However, the currently available treatments can cause unpredictable results and can incur potentially serious side effects [8]. PD patients show a heterogeneity of pathophysiology and clinical presentations, [9] which allows for the categorization of sub-types of PD [10]. PD patients also show heterogeneity in their response to various treatments, resulting in responders and non-responders to treatments [10]. This highlights the need to understand how different clinical sub-types of PD respond to treatment as well as the need to develop safe and effective treatments.

Photobiomodulation (PBM) is a non-invasive and non-thermal light therapy that benefits many health conditions and has recently been demonstrated to alleviate symptoms of neurological diseases [11]. PBM therapy uses wavelengths of red or near infrared light to penetrate tissue and affect cellular metabolism [12]. PBM therapy directly effects neuronal cellular metabolism and results in an increase in ATP production in the mitochondrial electron transport chain and down-regulation of proinflammatory cytokines, thereby increasing cellular energy and cell survival [13]. Animal PD models suggest that PBM is a potential adjunct therapy for PD [14]. Transcranial PBM (tPBM) has also been shown to immediately effect brain waves [15] and recently has been shown to improve cognitive function in patients with dementia [16] and chronic traumatic encephalopathy (CTE) [17]. Clinical trials of tPBM on PD patients, although limited, are encouraging, with a recent proof-of-concept waitlist study showing clinical improvements in PD symptoms [18].

The objective of the study reported here used the first wave of data from a triple-blind clinical study to evaluate the effect of tPBM on specific, clinically relevant PD motor symptoms, using a post-hoc analysis of sub-scores of MDS-UPDRS-III (motor domain). MDS-UPDRS-III sub-scores may more accurately reflect changes in PD motor symptoms than the total score.

## 2. Materials and Methods

**Figure 1:**
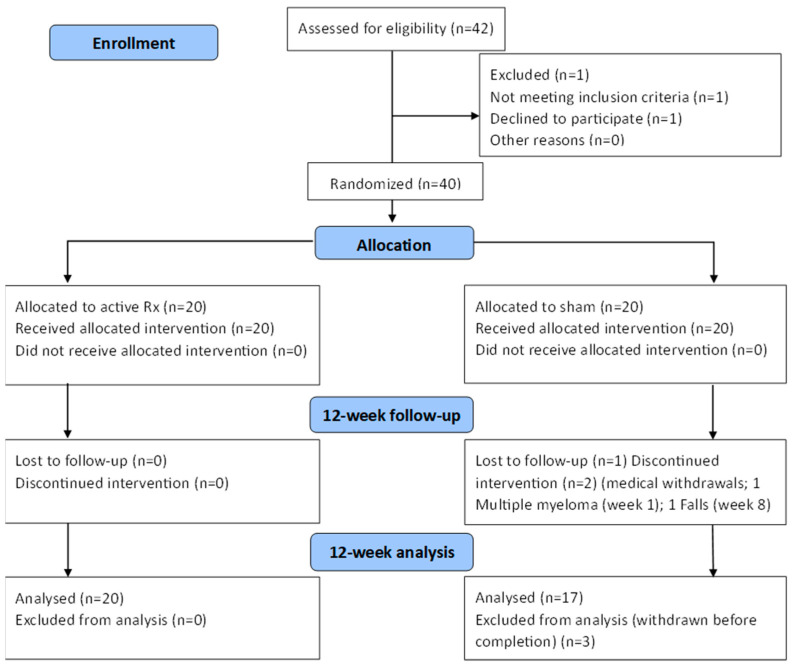
Consort Flow Diagram. Withdrawals: falls week 8, multiple myeloma week 1.

The study was approved by the Sydney Adventist Health Human Ethics Research Committee, approval number (2019-032). All patients/participants provided written informed consent to participate in this study. The study was registered with ANZCTR, registration identifier (12621001722886). The study design is shown in Figure 1.

The protocol used in this study has been described previously [19]. Briefly, the study was a triple-blind [20], randomized placebo-controlled trial (RCT), conducted over 24 weeks entirely remotely, with online, rather than in person face-to-face contact with trial participants. Conducting the study remotely was dictated by the SARS-CoV-2 pandemic and government constraints that were in place as public orders dictated the remote nature of the trial. Participants, assessors, and data analysts were blinded to active versus sham treatment. One participant liaison researcher was not blinded to the trial to provide continual technical and administrative assistance to participants.

Forty participants (20 male, 20 female) previously diagnosed by a neurologist with idiopathic PD (Hoehn and Yahr stage I or II, 65 to 80 years-of-age) and selected subject to inclusion and exclusion criteria (Supplementary Table S1), were randomized to the treatment or sham group via an independent administrator and thereafter identified via a study identification number. Participants received their tPBM device (active or sham) by mail and were instructed on how to apply the treatment via internet-based video conferences to ensure correct device fitment and operation. The MDS-UPDRS-III was collected and documented using a visual assessment obtained via Zoom video link. Each participant had a ‘carer’ that was able to manipulate the camera to ensure that the assessor had an optimal view. Each participant was re-assessed by the same assessor.

**Figure 2:**
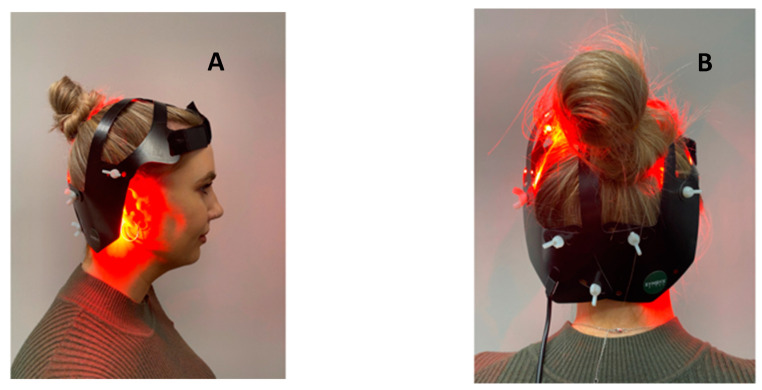
Images of the SYMBYX Neuro tPBM helmet. (**A**) Lateral view; (**B**) Posterior view.

The tPBM protocol consisted of 12 weeks of 24-min sessions, six times per week. The treatment group received tPBM with a SYMBYX Neuro helmet, purposely designed for treating symptoms of PD (Figure 2). It consisted of 40 diodes that delivered 12 min of red light (20 × 635 nm LEDs) followed by 12 min of infrared light (20 × 810 nm LEDs). A total of 37.44 and 19.44 joules were delivered from each of 20 diodes, providing a total of 1137 joules administered each session. The sham device was identical but delivered no therapeutic light. The sham group was told that light was infrared and could not be seen [19].

Outcome measures were assessed remotely by a neurologist and physiotherapists trained in the administration of the MDS-UPDRS-III before receiving their helmet device and after 12 weeks of treatment. Video assessment using this tool has been found to be a valid method for assessing PD [21]. However, two items were removed from the MDS-UPDRS-III due to safety concerns when conducted remotely (3:3 rigidity and 3:12 postural imbalance). This modified version has also been validated for use both in person and when conducted via remote delivery [22,23].

Treatment safety was addressed continuously, with participants having access to medical advice via direct 24-h phone access. Participants were also contacted (via video conference and email) every two weeks or more frequently if requested by the participant. Medical or health concerns were recorded as a Suspected Adverse Event (SAE). Each SAE was assessed by a team of two neurologists and the study coordinator. Any “side-effects” that the participants observed were also recorded. Treatment compliance was assessed by participant carers who monitored participant use of the PBM devices.

After 12 weeks of treatment, the RCT phase of the trial ended, and the cross-over phase of the trial began. Participants were unblinded and those in the sham group were offered 12 weeks of active treatment (cross-over phase) and those in the active group were untreated for 12 weeks (washout phase). Assessors and data analysts remained blinded to the treatment changes. Assessment was conducted at baseline, 12 weeks, and 24 weeks. This report describes the results from the first 12 weeks (RCT phase).

The first analysis was to assess the total MDS-UPDRS-III score and the second analysis was to investigate the individual components of the MDS-UPDRS-III. The individual items were combined into clinically relevant sub-scores: facial (items 3.1 and 3.2) upper-limb (items 3.4, 3.5, and 3.6), lower-limb (items 3.7, 3.8, and 3.9), gait (items 3.10, 3.11, 3.13, and 3.14) and tremor (items 3.15, 3.16, 3.17, and 3.18). Participants were also categorized as “responders” and “non-responders” based on an improvement in MDS-UPDRS-III scores of greater than or equal to 5, which corresponds to a moderate or large clinically important difference (CID) [24]. The study was pragmatic and there was no data to determine the power of the study. Significant changes in outcome measures were determined with paired t-tests using a *p* value of 0.05.

## 3. Results

Demographic details of the participants are shown in Supplementary Table S2. The safety of tPBM was established over the 12 weeks of the study, with no SAEs attributable to the treatment. The treatment was well tolerated, and compliance was excellent, with no withdrawals from the treatment group and three from the sham group (see Figure 1). The remote design of the study was easily managed, and participants stated that they used the device as prescribed.

The sham group at baseline had a higher average MDS-UPDRS-III score (mean = 26.0, sd = 13.81) than the treatment group (mean = 21.4, sd = 9.43), however, this difference was not significant. Total MDS-UPDRS-III scores improved significantly in both the treatment group (*p* = 0.011) and the sham group (*p* = 0.010) with a mean improvement of 23% and 24% above the baseline score in the treatment and sham groups, respectively (Table 1).

In the second analysis using sub-scores, there was no significant difference between the sham and treatment groups for any sub-score at baseline. At 12 weeks, the facial sub-score was significantly improved in the treatment group (*p* = 0.008) but not the sham group (*p* = 0.076), while the gait sub-score was significantly improved in the sham group (*p* = 0.046) but not the treatment group (*p* = 0.102). Both groups showed statistically significant improvement in the lower-limb sub-score (treatment *p* = 0.017; sham *p* = 0.007).

In the final analysis, when only responders were considered, all five clinically relevant sub-scores significantly improved in the treatment group (Table 1), with improvements of between 24% and 58%. The only significant improvement in the sham group was in the lower-limb sub-score.

## 4. Discussion

The first wave results of our study demonstrated a significant improvement in both the sham and active tPBM groups for total MDS-UPDRS-III scores. This dual improvement is most likely related to placebo effects in PD treatment trials, which are frequently documented [25,26]. Placebo responses in PD trials are predominately due to dopamine release [26], so this was not an unexpected finding [27].

While the gold standard assessment tool for PD is the MDS-UPDRS [28], the total score of the MDS-UPDRS-III may fail to meaningfully monitor motor changes and clinical improvements in some PD patients [29], due to the motor subtypes that can occur in PD [9,10]. Recent reexamination of motor symptoms in PD has suggested that symptoms have distinct patterns based on body location, which may manifest as differences in response to treatment [30,31]. When MDS-UPDRS-III items were grouped into sub-scores, responders to tPBM (approximately 70% of those receiving active treatment) showed a significant improvement in all MDS-UPDRS-III sub-scores. Although the use of MDS-UPDRS-III sub-group scores is a novel way of investigating PD, it may not inform on treatment specificity. Motor subtypes in PD have been recognized as clinically and pathophysiologically distinct [32], and MDS-UPDRS-III sub-scores may aid in uncovering specific treatment effects. In addition, analyses by responders and non-responders is often used in assessing medical devices and in pharmaceutical trials. Such analyses have been used in epilepsy [33], PD [34] and cognitive studies [35]. The identification of which patient characteristics can result in a positive response to treatment would help with further hypothesis generation and information regarding treatment effects.

There were several limitations in this study. The trial ran during the SARS-CoV-2 pandemic which necessitated investigators supervising participants remotely. Frequent internet video conferencing ensured that participants followed treatment protocols consistently. The sample size was small, but as a pragmatic study to assess feasibility of tPBM treatment of PD, it was sufficient to generate data to inform on future research. The 12-week treatment protocol was short for a progressive long-term disease; however, this was the first phase of a longer 24-week trial (reported elsewhere) that will yield more data and these initial promising results suggest that longer trials are warranted. While it is true that the MDS-UPDRS motor scores have been validated as a whole and the sub-score groupings have not, the use of the sub-score groupings in clinically relevant motor areas has generated useful data for further validation and studies.

## 5. Conclusions

This remotely run study is the first triple blind RCT assessing efficacy and safety of a novel transcranial PBM device for PD. It was found that tPBM was safe, well tolerated and improved specific motor symptoms for a majority of the sub-scores of the treatment cohort. Current treatments for PD provide limited long-term results, highlighting the need to examine new and less-researched therapies. The use of tPBM to treat symptoms of PD reflects an emerging application of light-based technologies to expand treatment options. The response of some participants to tPBM treatment in this study suggests that further research with a larger trial is needed to build an understanding of the application of tPBM to treat the symptoms of PD. Finally, the characteristics of PD patients who might respond positively to tPBM therapy requires further exploration. The results of this study are encouraging and suggest that tPBM can meaningfully improve individual motor signs of PD and be used as a safe and non-pharmaceutical adjunct treatment for the management of symptoms of PD.

## Figures and Tables

**Table 1:** UPDRS-MDS-III (modified) results of all participants and responders to active PBM treatment and sham treatment.

	Group	Baseline Mean (SD)	12-Week Mean (SD)	Mean Difference		Paired *t*-Test	
					Mean % Improvement	T Score	*p* Value
UPDRS scores for all participants (df: active = 19; sham = 17)							
Total score	Active	21.35 (9.43)	16.45 (9.48)	−4.90 (7.67)	23%	2.84	0.010 *
	Sham	26.00 (13.81)	20.47 (12.83)	−5.52 (7.93)	21%	2.85	0.011 *
Facial	Active	2.26 (1.44)	1.73 (1.66)	−0.53 (0.77)	23%	2.92	0.008 *
	Sham	2.24 (1.44)	1.88 (1.49)	−0.36 (0.93)	16%	1.56	0.138
Upper limb	Active	6.63 (3.53)	4.84 (3.82)	−1.79 (3.88)	27%	1.84	0.060
	Sham	7.24 (4.68)	6.59 (4.87)	−0.64 (3.37)	9%	0.79	0.440
Lower limb	Active	4.26 (2.51)	2.47 (2.38)	−2.26 (2.62)	53%	2.61	0.017 *
	Sham	6.24 (3.68)	3.88 (2.29)	−2.36 (3.16)	38%	3.04	0.007 *
Gait	Active	3.37 (1.54)	2.79 (1.87)	−0.58 (1.46)	17%	1.87	0.102
	Sham	5.00 (2.80)	3.65 (2.85)	−1.35 (2.57)	27%	2.16	0.046 *
Tremor	Active	4.84 (3.48)	4.11 (2.96)	−0.74 (2.58)	15%	0.51	0.229
	Sham	5.29 (5.59)	4.47 (4.45)	−0.82 (3.6)	16%	0.93	0.361
UPDRS scores for responders (df: active = 13; sham = 9)							
Total	Active	22.86 (10.39)	14.57 (8.87)	−8.29 (5.17)	36%	6.00	<0.001 *
score	Sham	29.80 10.39)	18.80 (14.31)	−11.00 (2.98)	37%	11.67	<0.001 *
Facial	Active	2.07 (1.38)	1.50 (1.51)	−0.57 (0.76)	28%	2.83	0.014 *
	Sham	2.10 (1.52)	1.50 (1.43)	−0.60 (0.97)	29%	1.97	0.081
Upper Limb	Active	7.07 (3.73)	4.29 (3.58)	−2.79 (3.89)	40%	2.68	0.019 *
	Sham	8.30 (5.31)	6.30 (5.25)	−2.00 (2.91)	24%	2.18	0.058
Lower Limb	Active	4.29 (2.73)	1.79 (2.12)	−2.50 (2.41)	58%	3.88	0.002 *
	Sham	7.60 (3.57)	3.70 (2.41)	−3.90 (2.57)	51%	4.82	0.001 *
Gait	Active	3.57 (1.40)	2.57 (1.79)	−1.00 (1.24)	28%	3.01	0.010 *
	Sham	5.60 (2.99)	3.60 (2.91	−2.00 (2.98)	36%	2.12	0.063
Tremor	Active	5.86 (3.39)	4.43 (3.03)	−1.43 (2.34)	24%	2.28	0.040 *
	Sham	6.20 (6.51)	3.70 (4.53)	−2.50 (3.63)	40%	2.18	0.057

SD = standard deviation; df = degrees of freedom; * = significant at *p* = 0.05.

## Supplementary Materials

The following supporting information can be downloaded at: https://www.mdpi.com/article/10.3390/jcm12082846/s1. Table S1: Inclusion and exclusion criteria; Table S2: Participant information.

## References

[1] Zeng X.S., Geng W.S., Jia J.J., Chen L., Zhang P.P. (2018). Cellular and Molecular Basis of Neurodegeneration in Parkinson Disease. Front. Aging Neurosci..

[2] Rocca W.A. (2018). The future burden of Parkinson’s disease. Mov. Dis..

[3] Dorsey E.R., Constantinescu R., Thompson J.P., Biglan K.M., Holloway R.G., Kieburtz K., Marshall F.J., Ravina B.M., Schifitto G., Siderowf A. (2007). Projected number of people with Parkinson disease in the most populous nations, 2005 through 2030. Neurology.

[4] Twelves D., Perkins K.S.M., Counsell C. (2003). Systematic review of incidence studies of Parkinson’s disease. Mov. Dis..

[5] Feigin V.L., Nichols E., Alam T., Bannick M.S., Beghi E., Blake N., Culpepper W.J., Dorsey E.R., Elbaz A., Ellenbogen R.G. (2019). Global, regional, and national burden of neurological disorders, 1990–2016: A systematic analysis for the Global Burden of Disease Study 2016. Lancet Neurol..

[6] Ruan X., Lin F., Wu D., Chen L., Weng H., Yu J., Wang Y., Chen Y., Chen X., Ye Q. (2021). Comparative Efficacy and Safety of Dopamine Agonists in Advanced Parkinson’s Disease with Motor Fluctuations: A Systematic Review and Network Meta-Analysis of Double-Blind Randomized Controlled Trials. Front. Neurosci..

[7] Limousin P., Foltynie T. (2019). Long-term outcomes of deep brain stimulation in Parkinson disease. Nat. Rev. Neurol..

[8] Zahoor I., Shafi A., Haq E. (2018). Pharmacological Treatment of Parkinson’s Disease. Parkinson’s Disease: Pathogenesis and Clinical Aspects.

[9] Hill E.J., Mangleburg C.G., Alfradique-Dunham I., Ripperger B., Stillwell A., Saade H., Rao S., Fagbongbe O., von Coelln R., Tarakad A. (2021). Quantitative mobility measures complement the MDS-UPDRS for characterization of Parkinson’s disease heterogeneity. Park. Relat. Disord..

[10] Campbell M.C., Myers P.S., Weigand A.J., Foster E.R., Cairns N.J., Jackson J.J., Lessov-Schlaggar C.N., Perlmutter J.S. (2020). Parkinson disease clinical subtypes: Key features & clinical milestones. Ann. Clin. Transl. Neurol..

[11] Hamblin M.R., Salehpour F. (2021). Photobiomodulation of the Brain: Shining Light on Alzheimer’s and Other Neuropathological Diseases. J. Alzheimer’s Dis..

[12] Heiskanen V., Hamblin M.R. (2018). Photobiomodulation: Lasers: Vs. light emitting diodes?. Photochem. Photobiol. Sci..

[13] Hamblin M.R., Liebert A. (2022). Photobiomodulation Therapy Mechanisms Beyond Cytochrome c Oxidase. Photobiomodulation Photomed. Laser Surg..

[14] Salehpour F., Hamblin M. (2020). Photobiomodulation for Parkinson’s Disease in Animal Models: A Systematic Review. Biomolecules.

[15] Zomorrodi R., Loheswaran G., Pushparaj A., Lim L. (2019). Pulsed Near Infrared Transcranial and Intranasal Photobiomodulation Significantly Modulates Neural Oscillations: A pilot exploratory study. Sci. Rep..

[16] Nizamutdinov D., Qi X., Berman M.H., Dougal G., Dayawansa S., Wu E., Yi S.S., Stevens A.B., Huang J.H. (2021). Transcranial near infrared light stimulations improve cognition in patients with dementia. Aging Dis..

[17] Naeser M.A., Martin P.I., Ho M.D., Krengel M.H., Bogdanova Y., Knight J.A., Hamblin M.R., Fedoruk A.E., Poole L.G., Cheng C. (2023). Transcranial Photobiomodulation Treatment: Significant Improvements in Four Ex-Football Players with Possible Chronic Traumatic Encephalopathy. J. Alzheimer’s Dis. Rep..

[18] Liebert A., Bicknell B., Laakso E.L., Heller G., Jalilitabaei P., Tilley S., Mitrofanis J., Kiat H. (2021). Improvements in clinical signs of Parkinson’s disease using photobiomodulation: A prospective proof-of-concept study. BMC Neurol..

[19] McGee C., Liebert A., Herkes G., Bicknell B., Pang V., McLachlan C.S., Kiat H. (2022). Protocol for randomized controlled trial to evaluate the safety and feasibility of a novel helmet to deliver transcranial light emitting diodes photobiomodulation therapy to patients with Parkinson’s disease. Front. Neurosci..

[20] Karanicolas P.J., Farrokhyar F., Bhandari M. (2010). Practical tips for surgical research: Blinding: Who, what, when, why, how?. Can. J. Surg..

[21] Tarolli C.G., Andrzejewski K., Zimmerman G.A., Bull M., Goldenthal S., Auinger P., O’Brien M., Dorsey E., Biglan K., Simuni T. (2020). Feasibility, Reliability, and Value of Remote Video-Based Trial Visits in Parkinson’s Disease. J. Parkinsons. Dis..

[22] Stillerova T., Liddle J., Gustafsson L., Lamont R., Silburn P. (2016). Remotely Assessing Symptoms of Parkinson’s Disease Using Videoconferencing: A Feasibility Study. Neurol. Res. Int..

[23] Abdolahi A., Scoglio N., Killoran A., Dorsey E.R., Biglan K.M. (2013). Potential reliability and validity of a modified version of the Unified Parkinson’s Disease Rating Scale that could be administered remotely. Park. Relat. Disord..

[24] Shulman L.M., Gruber-Baldini A.L., Anderson K.E., Fishman P.S., Reich S.G., Weiner W.J. (2010). The Clinically Important Difference on the Unified Parkinson’s Disease Rating Scale. Arch. Neurol..

[25] Goetz C.G., Wuu J., McDermott M.P., Adler C.H., Fahn S., Freed C.R., Hauser R.A., Olanow W.C., Shoulson I., Tandon P.K. (2008). Placebo response in Parkinson’s disease: Comparisons among 11 trials covering medical and surgical interventions. Mov. Dis..

[26] Lidstone S.C., Schulzer M., Dinelle K., Mak E., Sossi V., Ruth T.J., de la Fuente-Fernández R., Phillips A.G., Stoessl A.J. (2010). Effects of Expectation on Placebo-Induced Dopamine Release in Parkinson Disease. Arch. Gen. Psychiatry.

[27] Lidstone S.C. (2014). Great Expectations: The Placebo Effect in Parkinson’s Disease. Handbook of Experimental Pharmacology.

[28] Goetz C.G., Tilley B.C., Shaftman S.R., Stebbins G.T., Fahn S., Martinez-Martin P., Poewe W., Sampaio C., Stern M.B., Dodel R. (2008). Movement Disorder Society-sponsored revision of the Unified Parkinson’s Disease Rating Scale (MDS-UPDRS): Scale presentation and clinimetric testing results. Mov. Dis..

[29] Athauda D., Maclagan K., Budnik N., Zampedri L., Hibbert S., Aviles-Olmos I., Chowdhury K., Skene S.S., Limousin P., Foltynie T. (2019). Post hoc analysis of the Exenatide-PD trial-Factors that predict response. Eur. J. Neurosci..

[30] Bologna M., Paparella G., Fasano A., Hallett M., Berardelli A. (2020). Evolving concepts on bradykinesia. Brain.

[31] Askari A., Zhu B.J., Lyu X., Chou K.L., Patil P.G. (2022). Characterization and localization of upper and lower extremity motor improvements in STN DBS for Parkinson’s disease. Park. Rela Disord..

[32] Regnault A., Boroojerdi B., Meunier J., Bani M., Morel T., Cano S. (2019). Does the MDS-UPDRS provide the precision to assess progression in early Parkinson’s disease? Learnings from the Parkinson’s progression marker initiative cohort. J. Neurol..

[33] Dwivedi R., Tiwari P., Pahuja M., Dada R., Tripathi M. (2022). Anti-seizure medications and quality of life in person with epilepsy. Heliyon.

[34] Stubendorff K., Larsson V., Ballard C., Minthon L., Aarsland D., Londos E. (2014). Treatment effect of memantine on survival in dementia with Lewy bodies and Parkinson’s disease with dementia: A prospective study. BMJ Open.

[35] Standaert D.G., Boyd J.T., Odin P., Robieson W.Z., Zamudio J., Chatamra K. (2018). Systematic evaluation of levodopa-carbidopa intestinal gel patient-responder characteristics. NPJ Park. Dis..

