# Supplementary material for: A Randomized Placebo-Controlled Study of a Transcranial Photobiomodulation Helmet in Parkinson’s Disease: Post-Hoc Analysis of Motor Outcomes
Source: J Clin Med. 2023 Apr 13;12(8):2846. doi: 10.3390/jcm12082846 (PMC10146323; doi:10.3390/jcm12082846)
Supplement: Supplementary file 1 [file jcm-12-02846-s001.zip › jcm-2298229-supplementary.docx]

Supplementary Table S1. Inclusion and Exclusion Criteria

| **Supplementary Table 1:**  **Inclusion Criteria** | **Exclusion Criteria** |
| --- | --- |
| - Females and males aged 59–85 years, diagnosed with Idiopathic PD (by United Kingdom Brain Bank Criteria) with Modified Hoehn and Yahr Stage I–III during ON periods. - 3 weeks of stable anti-PD medications, sufficient space (around 9 m2) to be able to perform motor assessments. - Suitable and sufficiently fast home-based internet connection for uninterrupted video calls and video Conferencing. - Knowledge (self or carer) of using a phone and/or tablet applications on either IOS or Android platforms. - Attendance of a “carer” “support” person during each Zoom meeting and during all participant treatment sessions. | - Are not capable of self-care. - History of significant psychotic episode(s), suicidal ideation, or attempted suicide within the previous 12 months - Take potentially photosensitizing medications, in particular imipramine, hypericum, phenothiazine, lithium, chloroquine, hydrochlorothiazide, or tetracycline. - Have history of structural brain disease, active epilepsy, stroke, factors affecting gait performance and stance unrelated to PD, such as due to severe joint disease, orthopaedic injuries, weakness, peripheral neuropathy with proprioceptive deficits, severe peripheral vascular occlusive disease, severe musculoskeletal disorders, uncorrected vision, vestibular problems or other acute illness or severe condition that would: Preclude the use of PBM therapy, or place the patient at risk during evaluation of their PD, or interfere with the evaluation of their PD. - Are currently participating in other clinical trials, including treatment of PD. - Are currently using any form of self-administered light therapy. - Have evidence of severe and unstable dysautonomia. - Have significant cardiac disease. |

Supplementary Table S2. Participant information

| **Participant** | **Gender** | **Age** | **Time since diagnosis (years)** | **Medication**  **(L/S)** | **LEDD** |
| --- | --- | --- | --- | --- | --- |
| Sham |  |  |  |  |  |
| 1 | M | 80 | 14 | L | 800mg |
| 4 | F | 71 | 5 | L | 200mg |
| 5 | F | 75 | 6 | L | 450mg |
| 6 | M | 69 | 5 | L | 700mg |
| 8 | F | 78 | 6 | L | 850mg |
| 12 | F | 61 | 3 | L | 100mg |
| 14 | M | 74 | 17 | L,P | 600mg |
| 15 | M | 64 | 5 | L | 400mg |
| 19 | F | 69 | 5 | L | 900mg |
| 20 | M | 73 | 6 | L | 600mg |
| 21 | F | 65 | 2 | L | 300mg |
| 23 | M | 66 | 6 | L | 400mg |
| 27 | M | 69 | 10 | L | 800mg |
| 29 | F | 75 | 5 | L | 200mg |
| 30 | M | 72 | 2 | L | 200mg |
| 32 | M | 71 | 1 | L | NA |
| 33 | F | 78 | 11 | L | 800mg |
| 35 | M | 70 | 1 | L | NA |
| 36 | F | 71 | 3 | L | 400mg |
| 38 | F | 66 | 3 | L | 300mg |
| Mean sham | 10xF; 10xM | 70.9 | 5.8 |  |  |
|  |  |  |  |  |  |
| Active |  |  |  |  |  |
| 2 | M | 79 | 7 | L | 600mg |
| 3 | F | 67 | 3 | L | 300mg |
| 7 | M | 77 | 5 | L | 450mg |
| 9 | M | 67 | 6 | L | NA |
| 10 | M | 72 | 3 | L | 700mg |
| 11 | F | 77 | 5 | L | 300mg |
| 13 | M | 79 | 10 | P | 100mg |
| 16 | F | 72 | 2 | L | 300mg |
| 17 | F | 76 | 4 | L | 300mg |
| 18 | F | 78 | 1 | L | 300mg |
| 22 | M | 69 | 5 | L | 400mg |
| 24 | M | 69 | 4 | L | 300mg |
| 25 | F | 72 | 1 | nil |  |
| 26 | M | 61 | 1 | L | 300mg |
| 28 | M | 79 | 3 | L | 450mg |
| 31 | M | 75 | 5 | L | 400mg |
| 34 | F | 77 | 5 | nil |  |
| 37 | F | 78 | 4 | L | 150mg |
| 39 | F | 73 | 3 | P | 200mg |
| 40 | F | 69 | 10 | L | 300mg |
| Mean active | 10xF 10xM | 73.3 | 4.4 |  |  |
|  |  |  |  |  |  |
| Mean | 20xF 20xM | 72.0 | 5.1 |  |  |

L: L-dopa. P: pramipexole. LEDD: L-dopa equivalent daily dose. NA: data not available.
